# 2-Methyl-3-{2-nitro-1-[2-(prop-2-yn-1-yl­oxy)phen­yl]eth­yl}-1*H*-indole

**DOI:** 10.1107/S1600536811036907

**Published:** 2011-09-17

**Authors:** P. Narayanan, K. Sethusankar, K. Ramachandiran, P. T. Perumal

**Affiliations:** a Department of Physics, RKM Vivekananda College (Autonomous), Chennai 600 004, India; b Organic Chemistry Division, Central Leather Research Institute, Adyar, Chennai 600 020, India

## Abstract

In the title compound, C_20_H_18_N_2_O_3_, the indole unit is essentially planar, with a maximum deviation of 0.0197 (18) Å for the N atom and forms a dihedral angle of 78.09 (9)° with the propyne-subsituted phenyl ring. The propyne group is almost linear, the C—C C angle being 176.5 (2)°, and is also in the flagpole position on the O atom. In the crystal, mol­ecules are linked *via* N—H⋯O and C—H⋯O inter­molecular hydrogen bonds involving the nitro-group O atoms as acceptors.

## Related literature

For general backround to indoles, see: Gribble (1996 ▶); Mathiesen *et al.* (2005 ▶). For related structures, see: Narayanan *et al.* (2011 ▶); Ranjith *et al.* (2010 ▶). For bond-length distortions, see: Allen (1981 ▶).
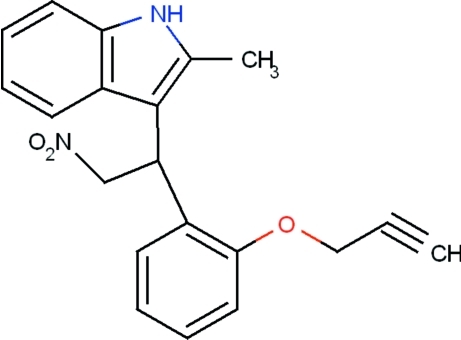


## Experimental

### 

#### Crystal data

C_20_H_18_N_2_O_3_
*M* *_r_* = 334.36
Tetragonal, 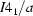

*a* = 23.3474 (7) Å
*c* = 12.8536 (7) Å
*V* = 7006.5 (5) Å^3^
*Z* = 16
Mo *K*α radiation
μ = 0.09 mm^−1^
*T* = 295 K
0.30 × 0.25 × 0.20 mm

#### Data collection

Bruker Kappa APEXII diffractometer
31091 measured reflections
3954 independent reflections
2629 reflections with *I* > 2σ(*I*)
*R*_int_ = 0.034

#### Refinement

*R*[*F* ^2^ > 2σ(*F* ^2^)] = 0.049
*wR*(*F* ^2^) = 0.149
*S* = 1.03
3954 reflections
231 parameters
H atoms treated by a mixture of independent and constrained refinement
Δρ_max_ = 0.35 e Å^−3^
Δρ_min_ = −0.30 e Å^−3^

### 

Data collection: *APEX2* (Bruker, 2008 ▶); cell refinement: *SAINT* (Bruker, 2008 ▶); data reduction: *SAINT*; program(s) used to solve structure: *SHELXS97* (Sheldrick, 2008 ▶); program(s) used to refine structure: *SHELXL97* (Sheldrick, 2008 ▶); molecular graphics: *ORTEP-3* (Farrugia, 1997 ▶) and *Mercury* (Macrae *et al.*, 2008 ▶); software used to prepare material for publication: *SHELXL97* and *PLATON* (Spek, 2009 ▶).

## Supplementary Material

Crystal structure: contains datablock(s) global, I. DOI: 10.1107/S1600536811036907/rk2291sup1.cif

Structure factors: contains datablock(s) I. DOI: 10.1107/S1600536811036907/rk2291Isup2.hkl

Supplementary material file. DOI: 10.1107/S1600536811036907/rk2291Isup3.cml

Additional supplementary materials:  crystallographic information; 3D view; checkCIF report

## Figures and Tables

**Table 1:** Hydrogen-bond geometry (Å, °)

*D*—H⋯*A*	*D*—H	H⋯*A*	*D*⋯*A*	*D*—H⋯*A*
N1—H1*A*⋯O2^i^	0.86	2.14	2.997 (2)	173
C11—H11*A*⋯O1^ii^	0.97	2.52	3.433 (3)	157
C15—H15⋯O1^iii^	0.93	2.57	3.315 (3)	137

Symmetry codes: (i) 
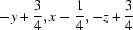
; (ii) 
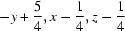
; (iii) 
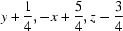
.

**Fig. 1.**
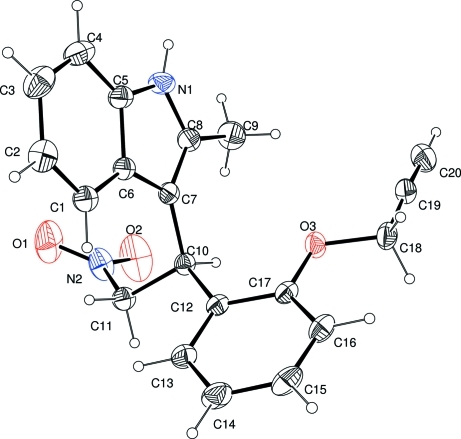
The molecular structure of the title compound with the atom numbering scheme. Displacement ellipsoids are drawn at 30% probability level. H atoms are present as small spheres of arbitary radius.

**Fig. 2.**
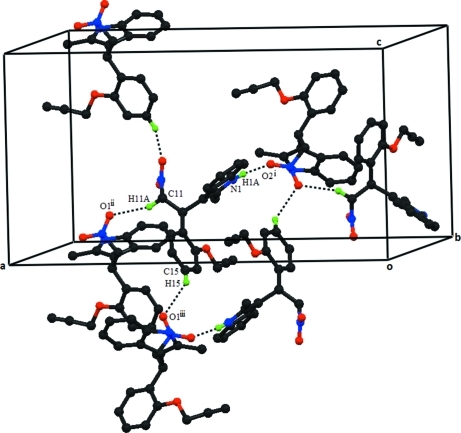
The packing arrangement of the title compound viewed down a axis. Dashed lines indicates the N—H···O and bifurcated C—H···O intermolecular hydrogen bonds. Symmetry codes as in the Table 1.

## supplementary crystallographic information

### Comment

Indole is a common motif for drug target and as such, of new diversity–tolerant routes to this previleged biological scaffold continues to be of significant benefit (Gribble, 1996) and forms the basis of a wide variety of drugs, including the anti–inflammatory agent indomethacin, reserpine and sumatriptan. Indole derivatives are identified as interfering with a *G* protein–independent signalling pathway of the *CRTH*2 receptor (Mathiesen *et al.*, 2005). As a part of our studies, we report herein the crystal structure of the title compound, which comprises the bicycle indole moiety, propyne subsituted phenyl ring and nitro methane group, as illustrated in (Fig. 1).

In the title compound, C_20_H_18_N_2_O_3_, the indole bicycle moiety (C1–C8/N1) is essentially planar with a maximum deviation of -0.0197 (18)Å for N1 atom. The indole moiety (C1–C8/N1) forms a dihedral angle of 78.09 (9)° with the propyne subsituted phenyl ring (C12–C17). In the indole ring system, the dihedral angle between the pyrrole ring (C5–C8/N1) and benzene ring (C1–C6) is 1.17 (10)°.

In the indole moiety, the endocyclic angles at C4 and C6 are contracted to 117.5 (2)° and 118.0 (17)°, respectively, while those at C2, C3 and C5 are expanded to 121.5 (2)°, 121.6 (3)° and 121.2 (3)°, respectively. This would appear to be a real effect caused by the fusion of the smaller pyrrole ring to the six–membered benzene ring, and the strain is taken up by the angular distortion rather than by bond–length distortions (Allen, 1981).

The angles around atom C10: [C7—C10—C12 = 113.88 (13)°, C7—C10—C11 = 110.41 (14)° and C12—C10—C11 = 109.95 (14)°] deviates significantly from ideal tetrahedral values which may be as a result of steric interactions between indole, nitromethane and propyne subsituted phenyl ring. The deviation of atom C10 from the indole moiety is -0.1066 (16)Å. The deviations of atom O3 from the phenyl ring (C12–C17) and propyne group (O3/C18/C19/C20) are 0.0504 (14)Å and 0.3088 (14)Å, respectively.

The oxygen subsituted propyne group is slightly twisted from the phenyl ring (C12–C17) which it is attached as evindenced by the torsion angle C16–C17–O3–C18 = 7.2 (3)°. The propyne group is almost linear, C18–C19≡C20 angle being 176.5 (2)°, and is also in the flagpole position on O3 atom. The title compound exhibits structural similarities with the already reported related structures (Narayanan *et al.*, 2011; Ranjith *et al.*, 2010).

In the crystal packing, molecules are linked *via* N—H···O and bifurcated C—H···O intermolecular hydrogen bonds involving the nitro group O atoms as acceptors (Table 1). The symmetry codes are: (i) -*y*+3/4, *x*-1/4, -*z*+3/4; (ii) -*y*+5/4, *x*-1/4, *z*-1/4; (iii) *y*+1/4, -*x*+5/4, *z*-3/4. The packing view of the title compound is shown in (Fig. 2).

### Experimental

To the nitroalkene (1.74 mmol) in water (10 ml) was added KHSO_4_ (30 mol%) and the mixture was stirred for 5 minutes. 1–Ethyl–indole (1.74 mmol) was added to the mixture and the stirring was continued following the progress of the reaction by *TLC*. After completion of the reaction, the reaction mixture was extracted with ethyl acetate (3× 10 ml), dried over anhydrous sodium sulfate, filtered, concentrated under reduced pressure and the residue was column chromatographed over silica gel using *Et*O*Ac* : Petroleum ether (1.5 : 8.5) as eluent to get the pure product.

### Refinement

The hydrogen atoms were placed in calculated positions with C—H = 0.89Å to 0.98Å, N—H = 0.86Å and refined in the riding model with fixed isotropic displacement parameters: *U*_iso_(H) = 1.5*U*_eq_(C)for methyl group and *U*_iso_(H) = 1.2*U*_eq_(C, N) for other groups.

In the crystal, solvent accessible void 42Å^3^ is found.

### Crystal data

**Table:**

C_20_H_18_N_2_O_3_	*D*_x_ = 1.268 Mg m^−^^3^
*M**_r_* = 334.36	Mo *K*α radiation, λ = 0.71073 Å
Tetragonal, *I*4_1_/*a*	Cell parameters from 3954 reflections
Hall symbol: -I 4ad	θ = 2.5–27.3°
*a* = 23.3474 (7) Å	µ = 0.09 mm^−^^1^
*c* = 12.8536 (7) Å	*T* = 295 K
*V* = 7006.5 (5) Å^3^	Block, brown
*Z* = 16	0.30 × 0.25 × 0.20 mm
*F*(000) = 2816	

### Data collection

**Table:**

Bruker Kappa APEXII diffractometer	2629 reflections with *I* > 2σ(*I*)
Radiation source: fine–focus sealed tube	*R*_int_ = 0.034
graphite	θ_max_ = 27.3°, θ_min_ = 2.5°
ω and φ scans	*h* = −30→30
31091 measured reflections	*k* = −30→30
3954 independent reflections	*l* = −16→16

### Refinement

**Table:**

Refinement on *F*^2^	Primary atom site location: structure-invariant direct methods
Least-squares matrix: full	Secondary atom site location: difference Fourier map
*R*[*F*^2^ > 2σ(*F*^2^)] = 0.049	Hydrogen site location: inferred from neighbouring sites
*wR*(*F*^2^) = 0.149	H atoms treated by a mixture of independent and constrained refinement
*S* = 1.03	*w* = 1/[σ^2^(*F*_o_^2^) + (0.061*P*)^2^ + 4.4075*P*] where *P* = (*F*_o_^2^ + 2*F*_c_^2^)/3
3954 reflections	(Δ/σ)_max_ = 0.001
231 parameters	Δρ_max_ = 0.35 e Å^−^^3^
0 restraints	Δρ_min_ = −0.30 e Å^−^^3^

### Special details

**Table:**

Geometry. All s.u.'s (except the s.u. in the dihedral angle between two l.s. planes) are estimated using the full covariance matrix. The cell s.u.'s are taken into account individually in the estimation of s.u.'s in distances, angles and torsion angles; correlations between s.u.'s in cell parameters are only used when they are defined by crystal symmetry. An approximate (isotropic) treatment of cell s.u.'s is used for estimating s.u.'s involving l.s. planes.
Refinement. Refinement of *F*^2^ against ALL reflections. The weighted *R*–factor *wR* and goodness of fit *S* are based on *F*^2^, conventional *R*–factors *R* are based on *F*, with *F* set to zero for negative *F*^2^. The threshold expression of *F*^2^ > σ(*F*^2^) is used only for calculating *R*–factors(gt) *etc*. and is not relevant to the choice of reflections for refinement. *R*–factors based on *F*^2^ are statistically about twice as large as those based on *F*, and *R*–factors based on ALL data will be even larger.

### Fractional atomic coordinates and isotropic or equivalent isotropic displacement parameters (Å^2^)

**Table:**

	*x*	*y*	*z*	*U*_iso_*/*U*_eq_	
C1	0.57180 (8)	0.58359 (8)	0.28887 (15)	0.0561 (5)	
H1	0.6050	0.5907	0.2511	0.067*	
C2	0.54456 (11)	0.62723 (10)	0.34038 (18)	0.0739 (6)	
H2	0.5595	0.6641	0.3365	0.089*	
C3	0.49531 (12)	0.61764 (12)	0.39812 (19)	0.0837 (7)	
H3	0.4784	0.6480	0.4335	0.100*	
C4	0.47114 (10)	0.56413 (12)	0.40392 (16)	0.0746 (6)	
H4	0.4379	0.5577	0.4421	0.090*	
C5	0.49805 (8)	0.52014 (9)	0.35072 (14)	0.0567 (5)	
C6	0.54906 (7)	0.52835 (8)	0.29372 (12)	0.0471 (4)	
C7	0.56448 (7)	0.47343 (7)	0.25110 (12)	0.0456 (4)	
C8	0.52332 (8)	0.43530 (9)	0.28289 (14)	0.0574 (5)	
C9	0.51646 (12)	0.37274 (10)	0.2625 (2)	0.0854 (7)	
H9A	0.4937	0.3559	0.3167	0.128*	
H9B	0.5535	0.3548	0.2610	0.128*	
H9C	0.4978	0.3673	0.1967	0.128*	
C10	0.61764 (7)	0.45897 (7)	0.19019 (12)	0.0461 (4)	
H10	0.6150	0.4185	0.1703	0.055*	
C11	0.67081 (8)	0.46567 (9)	0.25835 (14)	0.0556 (5)	
H11A	0.7049	0.4568	0.2182	0.067*	
H11B	0.6738	0.5049	0.2826	0.067*	
C12	0.62457 (7)	0.49375 (7)	0.09048 (12)	0.0466 (4)	
C13	0.66337 (9)	0.53799 (9)	0.07902 (15)	0.0608 (5)	
H13	0.6877	0.5469	0.1339	0.073*	
C14	0.66695 (11)	0.56947 (10)	−0.01207 (17)	0.0747 (6)	
H14	0.6936	0.5989	−0.0182	0.090*	
C15	0.63101 (11)	0.55691 (10)	−0.09283 (17)	0.0741 (6)	
H15	0.6327	0.5785	−0.1536	0.089*	
C16	0.59234 (10)	0.51273 (9)	−0.08517 (14)	0.0627 (5)	
H16	0.5681	0.5044	−0.1406	0.075*	
C17	0.58959 (8)	0.48052 (8)	0.00541 (13)	0.0486 (4)	
C18	0.52168 (9)	0.41607 (9)	−0.06941 (14)	0.0626 (5)	
H18A	0.5476	0.4081	−0.1266	0.075*	
H18B	0.4952	0.4458	−0.0912	0.075*	
C19	0.49038 (9)	0.36474 (10)	−0.04196 (16)	0.0655 (5)	
C20	0.46475 (12)	0.32315 (15)	−0.0253 (2)	0.0902 (8)	
N1	0.48348 (7)	0.46368 (8)	0.34151 (12)	0.0659 (5)	
H1A	0.4536	0.4482	0.3687	0.079*	
N2	0.66652 (9)	0.42633 (9)	0.34832 (17)	0.0796 (6)	
O1	0.66570 (12)	0.44706 (10)	0.43438 (16)	0.1362 (10)	
O2	0.66138 (13)	0.37606 (8)	0.3327 (2)	0.1423 (10)	
O3	0.55338 (6)	0.43482 (6)	0.01938 (9)	0.0586 (4)	
H20	0.4442 (13)	0.2919 (12)	−0.011 (2)	0.118 (11)*	

### Atomic displacement parameters (Å^2^)

**Table:**

	*U*^11^	*U*^22^	*U*^33^	*U*^12^	*U*^13^	*U*^23^
C1	0.0576 (11)	0.0581 (11)	0.0526 (10)	0.0033 (9)	−0.0041 (9)	0.0056 (9)
C2	0.0856 (16)	0.0634 (13)	0.0727 (14)	0.0126 (11)	−0.0077 (12)	0.0021 (11)
C3	0.0949 (18)	0.0863 (17)	0.0700 (15)	0.0366 (14)	−0.0004 (13)	−0.0033 (13)
C4	0.0628 (13)	0.1061 (19)	0.0551 (12)	0.0235 (13)	0.0091 (10)	0.0114 (12)
C5	0.0506 (10)	0.0771 (13)	0.0423 (9)	0.0048 (9)	−0.0010 (8)	0.0119 (9)
C6	0.0461 (9)	0.0606 (10)	0.0346 (8)	0.0030 (8)	−0.0055 (7)	0.0109 (7)
C7	0.0482 (9)	0.0536 (10)	0.0351 (8)	−0.0032 (7)	−0.0042 (7)	0.0098 (7)
C8	0.0602 (11)	0.0666 (12)	0.0453 (9)	−0.0122 (9)	−0.0003 (8)	0.0121 (9)
C9	0.1018 (18)	0.0704 (15)	0.0841 (16)	−0.0319 (13)	0.0091 (14)	0.0090 (12)
C10	0.0505 (9)	0.0473 (9)	0.0405 (8)	0.0002 (7)	−0.0030 (7)	0.0045 (7)
C11	0.0536 (10)	0.0630 (11)	0.0503 (10)	0.0055 (9)	−0.0033 (8)	0.0066 (9)
C12	0.0496 (9)	0.0515 (9)	0.0387 (8)	0.0034 (7)	0.0057 (7)	0.0033 (7)
C13	0.0667 (12)	0.0661 (12)	0.0497 (10)	−0.0107 (9)	0.0081 (9)	0.0049 (9)
C14	0.0923 (16)	0.0709 (14)	0.0610 (13)	−0.0162 (12)	0.0210 (12)	0.0106 (11)
C15	0.1057 (18)	0.0701 (13)	0.0466 (11)	0.0043 (12)	0.0202 (11)	0.0167 (10)
C16	0.0816 (14)	0.0664 (12)	0.0400 (10)	0.0116 (11)	0.0039 (9)	0.0073 (9)
C17	0.0527 (10)	0.0539 (10)	0.0392 (8)	0.0083 (8)	0.0044 (7)	0.0031 (7)
C18	0.0674 (12)	0.0758 (13)	0.0447 (10)	0.0065 (10)	−0.0129 (9)	−0.0074 (9)
C19	0.0575 (12)	0.0849 (15)	0.0542 (11)	0.0005 (11)	−0.0083 (9)	−0.0130 (11)
C20	0.0810 (17)	0.109 (2)	0.0808 (17)	−0.0283 (17)	−0.0087 (13)	−0.0050 (16)
N1	0.0560 (9)	0.0874 (12)	0.0542 (9)	−0.0139 (9)	0.0098 (8)	0.0156 (9)
N2	0.0929 (14)	0.0688 (12)	0.0771 (13)	−0.0006 (10)	−0.0388 (11)	0.0223 (10)
O1	0.211 (3)	0.1348 (18)	0.0629 (11)	−0.0503 (17)	−0.0414 (14)	0.0353 (12)
O2	0.209 (3)	0.0599 (11)	0.158 (2)	0.0096 (13)	−0.0778 (19)	0.0308 (12)
O3	0.0645 (8)	0.0686 (8)	0.0428 (7)	−0.0091 (6)	−0.0115 (6)	0.0054 (6)

### Geometric parameters (Å, °)

**Table:**

C1—C2	1.371 (3)	C11—H11A	0.9700
C1—C6	1.396 (3)	C11—H11B	0.9700
C1—H1	0.9300	C12—C13	1.382 (3)
C2—C3	1.387 (3)	C12—C17	1.399 (2)
C2—H2	0.9300	C13—C14	1.385 (3)
C3—C4	1.373 (4)	C13—H13	0.9300
C3—H3	0.9300	C14—C15	1.367 (3)
C4—C5	1.384 (3)	C14—H14	0.9300
C4—H4	0.9300	C15—C16	1.374 (3)
C5—N1	1.367 (3)	C15—H15	0.9300
C5—C6	1.411 (2)	C16—C17	1.387 (2)
C6—C7	1.440 (3)	C16—H16	0.9300
C7—C8	1.372 (2)	C17—O3	1.373 (2)
C7—C10	1.506 (2)	C18—O3	1.429 (2)
C8—N1	1.368 (3)	C18—C19	1.447 (3)
C8—C9	1.492 (3)	C18—H18A	0.9700
C9—H9A	0.9600	C18—H18B	0.9700
C9—H9B	0.9600	C19—C20	1.160 (4)
C9—H9C	0.9600	C20—H20	0.89 (3)
C10—C12	1.526 (2)	N1—H1A	0.8600
C10—C11	1.528 (2)	N2—O2	1.197 (3)
C10—H10	0.9800	N2—O1	1.208 (3)
C11—N2	1.480 (3)		
			
C2—C1—C6	119.24 (19)	C10—C11—H11A	109.8
C2—C1—H1	120.4	N2—C11—H11B	109.8
C6—C1—H1	120.4	C10—C11—H11B	109.8
C1—C2—C3	121.5 (2)	H11A—C11—H11B	108.3
C1—C2—H2	119.2	C13—C12—C17	117.67 (16)
C3—C2—H2	119.2	C13—C12—C10	123.84 (16)
C4—C3—C2	121.1 (2)	C17—C12—C10	118.49 (15)
C4—C3—H3	119.4	C12—C13—C14	121.8 (2)
C2—C3—H3	119.4	C12—C13—H13	119.1
C3—C4—C5	117.5 (2)	C14—C13—H13	119.1
C3—C4—H4	121.3	C15—C14—C13	119.4 (2)
C5—C4—H4	121.3	C15—C14—H14	120.3
N1—C5—C4	130.19 (19)	C13—C14—H14	120.3
N1—C5—C6	107.22 (17)	C14—C15—C16	120.67 (19)
C4—C5—C6	122.6 (2)	C14—C15—H15	119.7
C1—C6—C5	118.00 (17)	C16—C15—H15	119.7
C1—C6—C7	135.28 (16)	C15—C16—C17	119.8 (2)
C5—C6—C7	106.71 (16)	C15—C16—H16	120.1
C8—C7—C6	106.82 (16)	C17—C16—H16	120.1
C8—C7—C10	125.92 (17)	O3—C17—C16	124.02 (17)
C6—C7—C10	127.11 (15)	O3—C17—C12	115.38 (14)
N1—C8—C7	109.02 (18)	C16—C17—C12	120.60 (18)
N1—C8—C9	119.85 (18)	O3—C18—C19	108.69 (16)
C7—C8—C9	131.1 (2)	O3—C18—H18A	110.0
C8—C9—H9A	109.5	C19—C18—H18A	110.0
C8—C9—H9B	109.5	O3—C18—H18B	110.0
H9A—C9—H9B	109.5	C19—C18—H18B	110.0
C8—C9—H9C	109.5	H18A—C18—H18B	108.3
H9A—C9—H9C	109.5	C20—C19—C18	176.5 (2)
H9B—C9—H9C	109.5	C19—C20—H20	178 (2)
C7—C10—C12	113.88 (13)	C5—N1—C8	110.22 (15)
C7—C10—C11	110.41 (14)	C5—N1—H1A	124.9
C12—C10—C11	109.95 (14)	C8—N1—H1A	124.9
C7—C10—H10	107.4	O2—N2—O1	123.0 (2)
C12—C10—H10	107.4	O2—N2—C11	119.0 (2)
C11—C10—H10	107.4	O1—N2—C11	117.9 (2)
N2—C11—C10	109.25 (15)	C17—O3—C18	116.90 (14)
N2—C11—H11A	109.8		
			
C6—C1—C2—C3	0.7 (3)	C7—C10—C12—C13	105.5 (2)
C1—C2—C3—C4	−1.5 (4)	C11—C10—C12—C13	−19.0 (2)
C2—C3—C4—C5	0.5 (3)	C7—C10—C12—C17	−74.2 (2)
C3—C4—C5—N1	−179.0 (2)	C11—C10—C12—C17	161.27 (16)
C3—C4—C5—C6	1.3 (3)	C17—C12—C13—C14	1.7 (3)
C2—C1—C6—C5	1.0 (3)	C10—C12—C13—C14	−178.07 (18)
C2—C1—C6—C7	179.47 (19)	C12—C13—C14—C15	0.4 (3)
N1—C5—C6—C1	178.18 (15)	C13—C14—C15—C16	−1.3 (4)
C4—C5—C6—C1	−2.0 (3)	C14—C15—C16—C17	0.1 (3)
N1—C5—C6—C7	−0.71 (19)	C15—C16—C17—O3	−178.42 (18)
C4—C5—C6—C7	179.09 (17)	C15—C16—C17—C12	2.1 (3)
C1—C6—C7—C8	−178.48 (19)	C13—C12—C17—O3	177.55 (16)
C5—C6—C7—C8	0.12 (18)	C10—C12—C17—O3	−2.7 (2)
C1—C6—C7—C10	5.8 (3)	C13—C12—C17—C16	−2.9 (3)
C5—C6—C7—C10	−175.64 (15)	C10—C12—C17—C16	176.84 (16)
C6—C7—C8—N1	0.52 (19)	C4—C5—N1—C8	−178.7 (2)
C10—C7—C8—N1	176.34 (15)	C6—C5—N1—C8	1.1 (2)
C6—C7—C8—C9	179.6 (2)	C7—C8—N1—C5	−1.0 (2)
C10—C7—C8—C9	−4.6 (3)	C9—C8—N1—C5	179.79 (18)
C8—C7—C10—C12	125.70 (18)	C10—C11—N2—O2	58.0 (3)
C6—C7—C10—C12	−59.3 (2)	C10—C11—N2—O1	−118.6 (2)
C8—C7—C10—C11	−110.05 (19)	C16—C17—O3—C18	7.2 (3)
C6—C7—C10—C11	64.9 (2)	C12—C17—O3—C18	−173.23 (16)
C7—C10—C11—N2	60.3 (2)	C19—C18—O3—C17	174.73 (16)
C12—C10—C11—N2	−173.18 (16)		

### Hydrogen-bond geometry (Å, °)

**Table:**

*D*—H···*A*	*D*—H	H···*A*	*D*···*A*	*D*—H···*A*
N1—H1A···O2^i^	0.86	2.14	2.997 (2)	173.
C11—H11A···O1^ii^	0.97	2.52	3.433 (3)	157.
C15—H15···O1^iii^	0.93	2.57	3.315 (3)	137.

Symmetry codes: (i) −*y*+3/4, *x*−1/4, −*z*+3/4; (ii) −*y*+5/4, *x*−1/4, *z*−1/4; (iii) *y*+1/4, −*x*+5/4, *z*−3/4.
